# Effects of Dietary Protein and Fat Contents on Renal Function and Inflammatory Cytokines in Rats with Adriamycin-Induced Nephrotic Syndrome

**DOI:** 10.1155/2011/945123

**Published:** 2011-05-24

**Authors:** Su Yeon Kim, A Young Lim, Su Kyung Jeon, In Seok Lee, Ryowon Choue

**Affiliations:** ^1^Department of Medical Nutrition, Graduate School of East-West Medical Science, Kyung Hee University, Seocheon-dong, Giheung-gu, Yongin, Gyeonggi-do 446-701, Republic of Korea; ^2^Research Institute of Medical Nutrition, Kyung Hee University, Heikidong, Seoul 130-701, Republic of Korea

## Abstract

The effects of dietary protein and fat on renal function-related blood and urine parameters, such as albumin, urinary protein,and inflammatory cytokines were investigated in adriamycin- (ADR) induced nephrotic syndrome rats. ADR (2 mg/kg BW) was injected i.p. weekly for six weeks to develop nephrotic syndrome; thereafter rats were fed low-protein/high-fat (LPHF) or high-protein/low-fat (HPLF) diets for five weeks. Renal function-related blood and urine parameters were measured before and after dietary intervention. Serum levels of albumin, TG, and creatinine were significantly higher in the LPHF group than in the HPLF group. Serum levels of albumin were low and urinary protein excretion protein was high in HPLF group. BUN and UUN levels were higher in the HPLF group than in the LPHF. Urinary excretion of creatinine was significantly higher in the HPLF group than in the LPHF group. Serum inflammatory cytokine levels did not differ between the two groups, however the levels of IL-6, TNF-**α**, and IL-13 in splenocyte supernatants were significantly higher in the LPHF group than in the HPLF group. We confirmed that protein and fat contents in diet affect renal function-related blood and urine parameters and splenocyte inflammatory cytokine levels in ADR-induced nephrotic syndrome rats.

## 1. Introduction

Nephrotic syndrome (NS) is a general symptom of renal disease and is associated with abnormal immune response [1–3]. Nephrotic syndrome shows symptoms of hypoalbuminemia, hyperlipidemia, and proteinuria. It has been reported that dietary protein and fat affect the symptoms of nephrotic syndrome. Chronic degenerative diseases such as hyperlipidemia, hypertension, atherosclerosis, and diabetes mellitus promote renal function decline. 

Animal study showed that hyperlipidemia could damage renal vascular endothelial cells and cause glomerular interstitial cell proliferation by increasing platelet aggregation and platelet-derived growth factor levels [4]. Glomerular interstitial cell proliferation increases low-density lipoprotein cholesterol (LDL-chol) and free radical generation [5]. High-fat-diet-induced obesity causes hyperlipidemia showing structural and functional damages such as increased glomerular filtration rate (GFR), increased renal blood flow (RBF), and renal hypertrophy, as shown in both animal models and humans [6, 7]. 

Excessive protein intake may cause and accelerate kidney damage, and restricting dietary protein could prevent a decline in renal function and effectively protect the kidneys in animals [8]. Clinical trials have yet to determine the efficiency of this protocol [9]. Restricting dietary protein in renal patients increases exposure to malnutrition risk compared to that of normal people [9, 10]. Nevertheless, a nutrition treatment used for NS patients in Korea only focuses on restricting protein [10, 11] 

Cytokines are polypeptides that serve as cell-to-cell messengers. In patients relapsed into nephrotic syndrome, cytokines secreted from T cells increase glomerular permeability to protein [12]. Several studies have shown that nephrotic syndrome is associated with the presence of circulating permeability factors and complex immune system disturbances [13]. It seems that T-cell activation is involved in NS pathogenesis; however, the roles of specific cytokines are not clear [14, 15]. A previous study reported that TNF-*α* and IL-6 levels increased significantly in the NS animal model [16]. Expressions of anti-inflammatory cytokines are changed significantly in animals and human with nephrotic syndrome. A previous study reported that IL-4 and IL-10 were able to downregulate the release of vascular permeability factor in NS patients [17]. 

Therefore, we investigated the effects of dietary protein and fat contents on renal function and inflammatory cytokine levels in adriamycin- (ADR-) induced nephrotic syndrome rats [1].

## 2. Materials and Methods

### 2.1. Study Design

After two weeks of adaptation in a metabolic cage, animals received weekly 1.0 mL intraperitoneal injections of 2 mg/kg BW adriamycin (ADR) for six weeks (*n* = 20), after which, nephrotic syndrome was confirmed though proteinuria. ADR-treated animals were then placed into one of two different diet groups, a low-protein high-fat diet group, LPHF, or a high protein-low fat diet group, HPLF for five weeks. Both diets contained equal amounts of calories to prevent effects due to the differences in calorie intake. 

Body weight and food intake were measured weekly. The food efficiency ratio (FER) was calculated according to the following formula: (weight gain (g)/day)/(amount of food consumed (kcal)/day). Blood samples were obtained via heart puncture, and the organs (heart, liver, kidney, and spleen) were weighed after the animal was sacrificed. Blood and urine were collected at 0 and five weeks after experimental diets were fed.

### 2.2. Animals and Diets

The experimental protocol was approved by the Animal Care and Use Review Committee (IACUC) of Kyung Hee University. A total of 20 four-week-old male Sprague-Dawley rats with body weights ranging from 150 to 170 g were purchased from SLC, Inc (Shizuoka, Japan). Rats were housed in polycarbonate cages in temperature-controlled rooms (22 ± 2°C), with a relative humidity of 55 ± 5%, and a 12-h light/dark cycle. The mice were fed a pellet chow diet and were given water ad libitum for an adaption period of two weeks. After two weeks, rats were injected intraperitoneally (i.p.) with 2 mg/kg BW adriamycin (ADR, Doxorubicin, Sigma St Louis USA, D1515) weekly and were given AIN93G pellets (Research diet, USA) and water ad libitum for six weeks to induce ADR-induced proteinuria, which has been well characterized as the experimental model for nephrotic syndrome [1]. 

After the six week of induction, rats received either a low-protein/high-fat diet (7% of calories derived from protein and 40% from fat) or a high-protein/low-fat diet (30% of calories from protein and 7% from fat) for the remaining five weeks.

### 2.3. Analysis of Blood Samples

A 12-hour overnight fasting blood was collected at 0 and five weeks after feeding experimental diet. Rats were lightly anesthetized with ethyl ether, and blood samples were taken by heart puncture. Blood samples were immediately collected in EDTA-containing tubes and serum-separating tubes (SSTs) to separate the plasma and serum. Blood was centrifuged at 3,000 rpm for 15 min at 4°C, and the top layer (serum) was stored at −70°C until use in assays for serum lipid profile (TG, T-Chol, LDL-C, and HDL-C), kidney function (BUN, creatinine; sCr), and serum albumin level. We used a solid-phase sandwich enzyme-linked immunosorbent assay (ELISA) to measure albumin levels. A colored product was produced by bounding enzyme following the addition of a substrate solution, and the absorbance was measured at 595 nm. Albumin concentration was calculated relative to a standard curve. Serum lipid levels were determined using commercial kits (Asan Co Ltd, Seoul, Korea), as were serum BUN and creatinine levels (BioAssay System, Hayward, USA).

### 2.4. Analysis of Urine Samples

For 24-hour urine collection, animals were housed in metabolic cages. During the urinary collection period, rats were restricted from food to avoid contaminating the urine. They were, however, allowed free access to water. Instruments used to collect urine were washed with 0.1 N HCl to prevent rot. Urine samples were centrifuged at 2,000 rpm for 15 min at room temperature, and the top layer was stored at −70°C until used to assay kidney function parameters (proteinuria, UUN, UCr). Urinary protein was measured using the Bradford method, which relies on protein binding to Coomassie Blue G-250 (BioRad no. 500-0201) dye. Urinary urea nitrogen (UUN) and urinary creatinine were determined using commercial kits (BioAssay System, Hayward, USA).

### 2.5. Hepatic Lipid Profile

After blood collection, a portion of the liver was frozen in liquid nitrogen and maintained at −70°C until assayed. Total lipid was extracted from liver using Folch's method. The extracted samples were dried under nitrogen gas, resolved in 2-propanol containing 10% Triton X-100 (wt : wt), and subjected to measurement of the lipid components. Triglyceride and total cholesterol concentration were determined using enzyme assay kits (Asan Pham Co., Seoul, Korea).

### 2.6. Clinical Parameters

Creatinine clearance (Ccr) was calculated according to the following formula [18]:

(1) $$\;\text{Ccr}\;\left(\text{mL}/\min\;\right)=\frac{\text{Ucr}\;\left(\text{mg}/\text{day}\right)}{\text{Scr}\;\left(\text{mg}/\text{mL}\right)\times 1,440\;\left(\min\;/\text{day}\right)},$$

where Ccr is creatinine clearance, Ucr is urinary creatinine, Scr is serum creatinine, and 1,440 = 24 h × 60 min .

### 2.7. Inflammatory Cytokine Profile

Proinflammatory (IL-1*β*, IL-6, and TNF-*α*) and anti-inflammatory (IL-4, IL-10 and IL-13) cytokines were measured in duplicate using Millipore's MILLIPLEX rat cytokine panel (Millipore, Billerica, Mass). Using multiplex technology, all cytokines in the panel were measured simultaneously. All assays were conducted according to the manufacturer's instructions. The plate was run on a Luminex 200 Instrument using Bio-Plex Manager 4.1 standard software (Bio-Rad Laboratories, Hercules, Calif). Raw fluorescence data were analyzed by the software using a five-parameter logistic method. The minimum detection concentrations were 1.8, 1.0, 0.4 and 6.3 pg/mL for IL-6, TNF-*α*, IL-4, and IL-13, respectively. The intra- and interassay precisions of the rat cytokine panel were 2.16–9.12% and 3.11–5.86%, respectively.

### 2.8. Primary Spleen Cells Culture

Spleens were isolated, and single-splenocyte suspensions were prepared and adjusted to 2 ×10^6^ cells/mL in RPMI 1640 supplemented with 10% FBS, 1% 5 × 10^−5^ M 2-mercaptoethanol, and 1% antibiotic antimycotic solution (Sigma). Cell suspensions were plated in 24-well flat-bottom culture plates at 1 mL per well in the presence of LPS (5 *μ*g/mL) and ConA (5 *μ*g/mL). Each culture was maintained for 24 h at 37°C and 5% humidity.

### 2.9. Statistical Analysis

All measurements were performed in duplicate, and statistical calculation was performed with SPSS statistical software for Windows version 13.0. All data are presented as mean ± SD. Differences in measured parameters between the experimental groups were analyzed using Student's *t*-test. The differences were considered to be significant when the *P*-value was less than  .05.

## 3. Results

### 3.1. Body Weight, Calorie Intake, Food Efficiency Ratio, and Organ Weights

The effects of dietary protein and fat contents on weight, calorie intake, the food efficiency ratio (FER), and organ weights are shown in Table 1. The baseline body weight of LPHF and HPLF diet groups were 374.3 ± 11.4 and 366.4 ± 16.4 g, respectively. Five weeks of feeding the experimental diets did not affect the final body weights (415.5 ± 13.0 g and 411.5 ± 12.1 g, resp.). Calorie intake (58.4 ± 0.5 and 58.0 ± 0.3 kcal/day, resp.) and FER (0.4 ± 0.1 and 0.5 ± 0.1, resp.) were not significantly different between the groups.

After feeding the experimental diets for five weeks, the average weights of liver, spleen, and heart did not differ between the groups. However average kidney weight of the HPLF group (3.4 ± 0.6 g) was significantly higher than that of the HPLF (2.4 ± 0.1 g) group (*P* < .05).

### 3.2. Blood and Hepatic Lipid Levels

**Figure 1:**
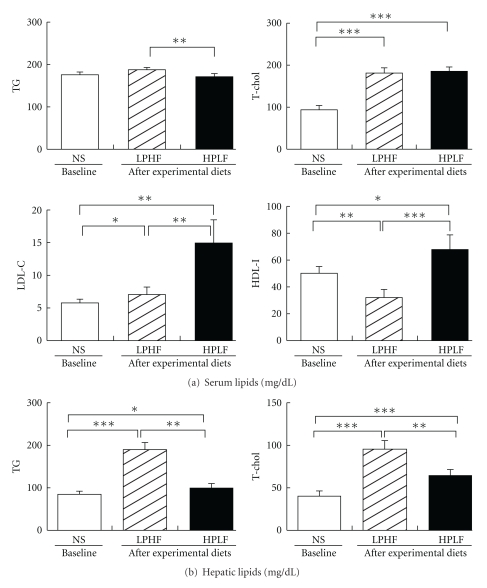
Serum and hepatic lipids. NS: weekly intraperitoneal injection of adriamycin (2 mg/kg BW) for six weeks. LPHF: weekly intraperitoneal injection of adriamycin (2 mg/kg BW) for 6 weeks thereafter a 7% protein and 40% fat diet for 5 weeks. HPLF: weekly intraperitoneal injection of adriamycin (2 mg/kg BW) for 6 weeks thereafter a 30% protein and 7% fat diet for 5 weeks.TG: triglyceride, T-chol: total-cholesterol, LDL-chol: LDL-cholesterol, HDL-chol: HDL cholesterol. *Statistically different between the LPHF and HPLF groups by student's *t*-test at *P* < .05, **P < .01, ***P < .001.

The effects of experimental diets on the levels of blood and hepatic lipids are shown in Figure 1. The serum triglyceride (TG) level in the LPHF group (186.9 ± 5.6 mg/dL) was significantly higher than that in the HPLF group (170.9 ± 6.4 mg/ dL) (*P* < .01); however, it did not differ from the baseline value (177.9 ± 5.3 mg/dL). The serum T-chol levels were not different between the groups, both of which (186.7 ± 8.8 mg/dL, and 182.0 ± 11.1 mg/dL, resp.) were significantly higher than the baseline value (94.1 ± 10.1 mg/dL, *P* < .001). The serum level of LDL-chol was significantly higher in the HPLF (15.0 ± 3.5 mg/dL) than in the LPHF group (7.5 ± 1.1 mg/dL), both of which were significantly higher than the baseline value (5.8 ± 0.5 mg/dL, *P* < .05). The serum HDL-chol levels were significantly lower in the LPHF group (32.4 ± 6.0 mg/dL) than in the HPLF group (68.1 ± 11.1 mg/dL) (*P* < .01), with that of the LPHF group being significantly lower than the baseline value (50.4 ± 4.5 mg/dL, *P* < .05). 

The hepatic TG contents were significantly higher in the LPHF group (189.1 ± 17.3 mg/dL) than those in the HPLF group (100.5 ± 9.4 mg/dL) (*P* < .01) both of which were significantly higher and lower than the baseline value, respectively (*P* < .001, *P* < .05). The hepatic contents of T-chol were also significantly higher in the LPHF group (95.8 ± 10.4 mg/dL) than in the HPLF group (64.4 ± 6.9 mg/dL) (*P* < .001) both of which were significantly higher than the baseline value (40.3 ± 6.2 mg/dL) (*P* < .05).

### 3.3. Blood and Urinary Clinical Parameters

**Figure 2:**
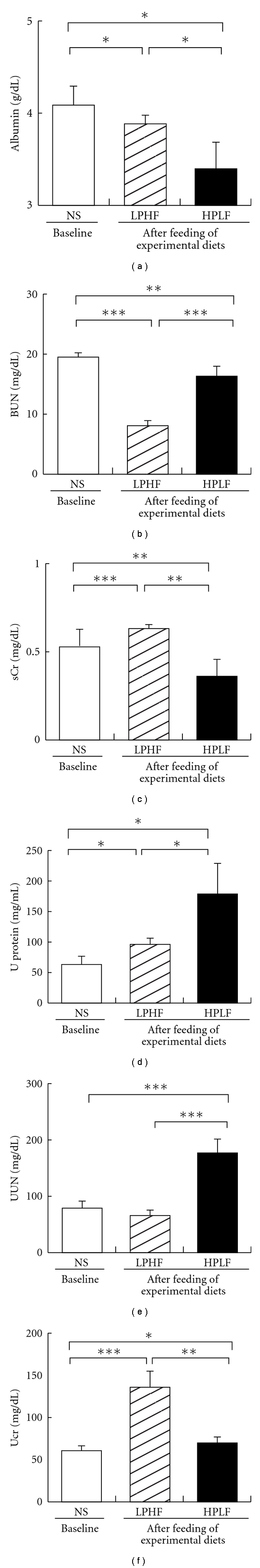
Blood and urinary clinical parameters. NS: weekly intraperitoneal injection of adriamycin (2 mg/kg BW) for six weeks. LPHF: weekly intraperitoneal injection of adriamycin (2 mg/kg BW) for 6 weeks thereafter a 7% protein and 40% fat diet for five weeks. HPLF: weekly intraperitoneal injection of adriamycin (2 mg/kg BW) for 6 weeks thereafter a 30% protein and 7% fat diet for five weeks. BUN : blood urea nitrogen, Scr: serum creatinine. UUN: urinary urea nitrogen, Ucr: urinary creatinine, Ccr: creatinine clearance. *Statistical difference between groups by Student's *t*-test at *P* < .05, **P < .01, ***P < .001.

The effects of dietary protein and fat contents on blood and urinary clinical parameters are shown in Figure 2. The serum level of albumin in the LPHF group (3.9 ± 0.1 g/dL) was higher that of the HPLF group (3.4 ± 0.3 g/dL) showing these values were significantly lower than the baseline value (4.1 ± 0.1 g/dL) (*P* < .05). The mean levels of BUN in the LPHF group was 8.3 ± 0.6 mg/dL and that of HPLF group was 16.1 ± 1.2 mg/dL, both groups were significantly lower than the baseline value (19.5 ± 0.7 mg/dL, *P* < .01). The sCr level in the LPHF group was 0.50 ± 0.05 mg/dL and that of the HPLF group was 0.36 ± 0.05 mg/dL, those of both groups were significantly higher and lower than the baseline values, respectively (*P* < .01). 

Urinary protein excretion in the HPLF group (178.6 ± 55.9 mg/24 h mL) was significantly higher than that in the LPHF group (95.7 ± 10.5 mg/24 h mL), and both were significantly higher than the baseline value (72.8 ± 9.6 mg/24 h mL, *P* < .05). The level of UUN in the HPLF group (178.3 ± 24.7 mg/dL) was higher than that of the LPHF group (66.3 ± 11.1 mg/dL) and the baseline value (81.0 ± 13.0 mg/dL) (*P* < .001). The Ucr level of the LPHF group was 135.8 ± 18.8 mg/dL, and that of the HPLF group was 71.2 ± 5.1 mg/dL (*P* < .001), both values were significantly higher than the baseline values (60.1 ± 6.1 mg/dL, *P* < .05). 

The creatinine clearance (Ccr) of the LPHF group was 1.4 ± 0.2 mL/min and that of the HPLF group was 1.4 ± 0.2 mL/min, showing a significant difference (*P* < .05). Ccr of both groups were significantly lower than the baseline values (2.13 ± 0.05 min/mL, *P* < .05) (data not shown).

### 3.4. Serum and Splenocyte Supernatant Levels of Inflammatory Cytokines

The effects of dietary protein and fat on serum inflammatory cytokine levels are shown in Table 2. The serum levels of neither the Proinflammatory cytokines (IL-1*β*, IL-6, and TNF-*α*) nor the anti-inflammatory cytokines (IL-4, IL-10, and IL-13) in the experimental groups did not differ significantly. Serum levels of IL-1*β* in LPHF group, and IL-6, TNF-*α* and IL-13 levels in HPLF group were significantly lower than each of the baseline values (*P* < .05). 

**Figure 3:**
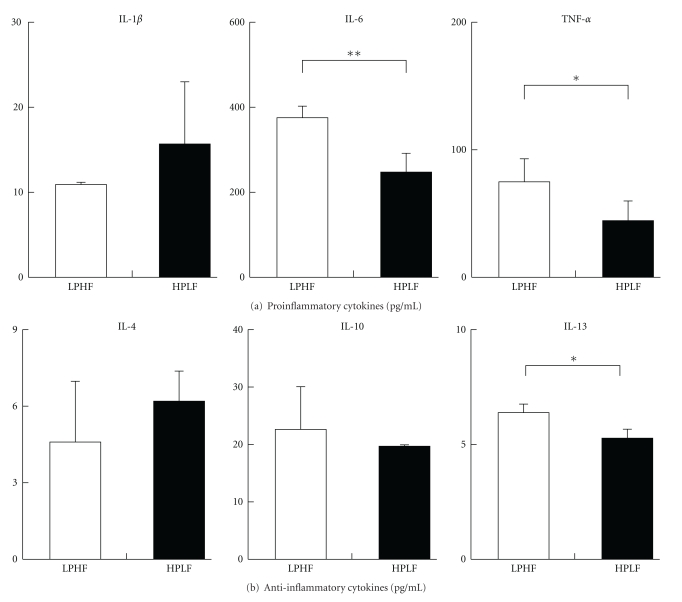
Inflammatory cytokines levels in splenocyte supernatant. LPHF: weekly intraperitoneal injection of adriamycin (2 mg/kg BW) for six weeks thereafter a 7% protein and 40% fat diet for five weeks. HPLF: weekly intraperitoneal injection of adriamycin (2 mg/kg BW) for six weeks thereafter a 30% protein and 7% fat diet for five weeks. *Statistical difference between the LPHF and HPLF groups by Student's *t*-test at *P* < .05, **P < .01.

The effects of dietary protein and fat composition on splenocyte supernatant levels of inflammatory cytokines are shown in Figure 3. The splenocyte supernatant levels of Proinflammatory cytokines, IL-6 and TNF-*α*, in the LPHF group (374.0 ± 27.0 pg/mL and 75.2 ± 17.7 pg/mL, resp.) were significantly higher than those of the HPLF group (245.5 ± 45.5 pg/mL, and 44.0 ± 15.2 pg/mL, resp.) (*P* < .05), whereas IL-1*β* in the splenocyte supernatant did not differ between the groups. The splenocyte supernatant level of the anti-inflammatory cytokine, IL-13, in the LPHF group (6.40 ± 0.36 pg/mL) was significantly higher than that of HPLF group (5.25 ± 0.43 pg/mL) (*P* < .05), whereas the IL-4 and IL-10 levels did not differ between the groups.

## 4. Discussion

This study was conducted to investigate the effects of dietary protein and fat contents on renal function and inflammatory cytokine levels in rats with nephrotic syndrome. We induced nephrotic syndrome to the rats by intraperitoneal injection of ADR (2 mg/kg BW) for six weeks, followed by a low-protein/high-fat (C : P : F = 53 : 7 : 40, LPHF) or a high-protein/low-fat (C : P : F = 63 : 30 : 7, HPLF) diets to the rats for five weeks. The main symptoms of nephrotic syndrome might be hypoalbuminemia, hyperlipidemia, and proteinuria [1–3]. First of all, we could observe that serum albumin levels decreased and serum lipid levels increased, as well as urinary protein increased. Hence, in accord with previous findings, these results ascertained the symptoms of nephrotic syndromes had been developed before the treatment of experimental diets. 

To prevent the effects of different amount of calories from the experimental diets, we offered isocalorie diets with different protein:fat ratios. Consequently, weight gain, calorie intake, and FER did not differ significantly between the two diet groups. 

In nephrotic syndrome rats, the kidney size might increase because of the damages from excessive urinary protein loss and inflammation. Previous studies reported that a high-protein diet caused the kidney damage more than a low-protein diet did [19, 20]. In our study, the kidney weights of the high-protein/low-fat diet group were significantly higher than those of low-protein/high-fat diet group. Renal hypertrophy may be developed by kidney overload due to the considerable protein in the HPLF diet.

Generally, it is well known that rats with nephrotic syndrome had significantly higher plasma lipid levels caused by variations of oncotic pressure due to proteinuria, compared with normal rats [21, 22]. It is also reported that hyperlipidemia in nephrotic syndrome can be more aggravated by abnormalities of various lipoproteins [23]. In patients with nephrotic syndrome, liver production and secretion of cholesterol-rich lipoproteins increased, and cholesterol removal in blood circulation decreased. It was proven that the overproduction of lipoproteins results in their accumulation in the livers of the rats with nephrotic syndrome [21, 24]. In this study, the serum levels of TG in the two experimental diet groups were similar to those at baseline, though the serum TG level of the HPLF group was significantly lower than those of the LPHF group. On the other hand, T-chol levels of both groups were higher than the baseline values, though the levels of the HPLF and LPHF groups were similar to one another.

Additionally, some studies have reported that hyperlipidemia is significant due to high serum TG and T-chol levels in high-fat diet [25, 26]. In this study, LDL-chol and HDL-chol of the LPHF group were higher than those at baseline. Therefore it is considered that the hyperlipidemia in LPHF group is significant due to the large portion of fat in diet. However, additional studies are needed to identify the mechanisms of increased LDL- and HDL-chol levels in high protein diet.

Generally, it is known that injury to podocyte foot processes in the kidney causes protein excretion into the urine [27]. In fact, proteinuria indicates a decline of renal function [28–30] and it is known that ADR injection causes proteinuria, as shown previously [2, 3, 29, 30]. In our study, the experimental diets were provided after inducing proteinuria by ADR injection. Urinary protein excretions after dietary treatments were significantly different and which were higher than that of baseline value. It is considered that the increased protein excretion due to high-protein diet is consistent with previous studies, and consequently the progress of nephrotic syndrome in HPLF group was more advanced than that of LPHF group.

 Serum albumin, BUN, sCr, UUN, Ucr, and Ccr are known as indicators about renal dysfunction [31–33]. As seen above, we could develop the symptoms of nephrotic syndrome in our animal model. Then, we could explore the effect of dietary protein and fat contents on renal function. Each diet advanced the progress of nephrotic syndrome, as evidenced by serum albumin and urinary protein levels compared with the values at baseline. It is considered that a lower serum albumin level in the HPLF group indicates that nephrotic syndrome progressed further in the HPLF group than in the LPHF group. After dietary intervention for 5 weeks, the BUN and UUN of both groups were lower than those at baseline. The BUN and UUN in the HPLF group were significantly higher than those of the LPHF group, indicating negative influence of high-protein diet on the renal function. The sCr and Ucr in the HPLF group were lower than baseline, and the levels of the LPHF group were higher than those of the HPLF group. Thus, the experimental diets have no influence on the levels of sCr and Ucr. The Ccr in the HPLF group was higher than baseline, while that of the LPHF group was lower than baseline. These results could be speculated that high protein in the HPLF diet might cause hyperfiltration, whereas high fat in the LPHF might cause hypofiltration [31, 32]. 

 Generally, nephrotic syndrome has been known to be associated with a variety of abnormalities in immune response. Thus, inflammatory cytokines in nephrotic syndrome are related to increased ROS production due to proteinuria [34]. In addition, a high-fat diet decreases immune responses due to lipid peroxidation in kidney [33, 34].

Previous studies have shown that the expressions of Proinflammatory cytokines, IL-6 and TNF-*α*, both increase in nephrotic syndrome [34–36]. The ADR injection affects renal function which is influenced by the diet, thereby influencing the levels of serum Proinflammatory cytokines. In this study, the levels of these cytokines in serum and in the splenocyte supernatant were measured in different dietary group. After feeding different diets, serum levels of inflammatory cytokines did not differ between the dietary groups. However levels of IL-6 and TNF-*α* in splenocyte supernatant were significantly lower in the HPLF group than in the LPHF group. 

The level of anti-inflammatory cytokine, IL-13 was also significantly lower in the HPLF group than that in the LPHF group. Carefully it can be said that splenocyte's function may be more damaged by a high-protein diet than by a low-protein diet. However, this result differs from those of previous studies [37–39]. The reasons of this discrepancy are not clear at present. Therefore we calculated the ratio of IL-6 to IL-13 in splenocyte supernatant as the balance between pro- and anti-inflammatory cytokines. The ratio of IL-6 to IL-13 in the HPLF group was higher than the value at the baseline, confirming an increase in Proinflammatory cytokines, while the ratio in the LPHF group was lower than the baseline value, showing a decrease in Proinflammatory cytokines (data are not shown).

This study found that a low-protein/high-fat diet may improve renal function in nephrotic syndrome as indicated by proteinuria, serum album level, BUN, and UUN. Furthermore, this diet might improve inflammatory state in nephritic syndrome rats through protecting splenocyte immune function. Accordingly, varying dietary protein and fat in nephrotic syndrome influence renal function and inflammatory state. Furthermore, we need to explore minutely the effects of various diet compositions on renal function in nephrotic syndrome animal model.

## Figures and Tables

**Table 1:** Body weights, food intakes, FER, and organ weights.

	LPHF^(1)^	HPLF^(2)^
Initial body weight (g)	374.3 ± 11.4	366.4 ± 16.4
Final body weight (g)	415.5 ± 13.0	411.5 ± 12.1
Weight gain (g)	41.2 ± 10.3	40.0 ± 8.5
Calories intake (kcal/day)	58.4 ± 0.5	58.0 ± 0.3
FER^(3)^	0.4 ± 0.1	0.5 ± 0.1

Liver (g)	12.2 ± 0.6	11.9 ± 1.0
Spleen (g)	0.7 ± 0.1	0.8 ± 0.2
Kidney (g)	2.6 ± 0.1	3.4 ± 0.6*
Heart (g)	1.2 ± 0.1	1.2 ± 0.1

^(1)^LPHF: weekly intraperitoneal injection of adriamycin (2 mg/kg BW) for 6 weeks thereafter a 7% protein and 40% fat diet for 5 weeks.

^(2)^HPLF: weekly intraperitoneal injection of adriamycin (2 mg/kg BW) for 6 weeks thereafter a 30% protein and 7% fat diet for 5 weeks.

^(3)^FER: food efficiency ratio: (weight gain (g))/(food consumed (kcal)/day).

*Statistical difference between the HPLF and LPHF groups by Student's *t*-test at *P* < .05.

**Table 2:** Serum levels of inflammatory cytokines.

	LPHF^(1)^	HPLF^(2)^
*Proinflammatory cytokines *(*pg/mL*)		
IL-1*β*	23.5 ± 1.1	29.3 ± 9.0
IL-6	113.4 ± 84.2	95.8 ± 23.5
TNF-*α*	3.0 ± 0.3	2.8 ± 0.2

*Anti-inflammatory cytokines *(*pg/mL*)		
IL-4	23.8 ± 2.4	25.8 ± 3.6
IL-10	3.1 ± 0.4	3.7 ± 0.9
IL-13	44.7 ± 4.4	52.4 ± 19.6

^(1)^LPHF: weekly intraperitoneal injection of adriamycin (2 mg/kg BW) for 6 weeks thereafter a 7% protein and 40% fat diet for five weeks.

^(2)^HPLF: weekly intraperitoneal injection of adriamycin (2 mg/kg BW) for 6 weeks thereafter a 30% protein and 7% fat diet for five weeks.

No statistical difference between the LPHF and HPLF groups by Student's *t*-test at *P* < .05.
